# Composite Hydrogel Modulates Intrinsic Immune-Cascade Neovascularization for Ocular Surface Reconstruction after Corneal Chemical Injury

**DOI:** 10.3390/gels9090676

**Published:** 2023-08-22

**Authors:** Jun Zhang, Kun Xi, Guohua Deng, Xi Zou, Peirong Lu

**Affiliations:** 1 Department of Ophthalmology, The First Affiliated Hospital of Soochow University, 188 Shizi Street, Suzhou 215000, China; nydczsyzj@163.com; 2 Department of Ophthalmology, Changzhou Third People’s Hospital, Changzhou Medical Center, Nanjing Medical University, 300 Lanlin North Road, Changzhou 213000, China; czsydgh@163.com (G.D.); 15851308890@163.com (X.Z.); 3 Department of Orthopedic Surgery, Orthopedic Institute, The First Affiliated Hospital of Soochow University, 188 Shizi Street, Suzhou 215000, China; sudaxk@163.com

**Keywords:** composite hydrogel, neutrophil extracellular traps, corneal alkali burn, neovascularization

## Abstract

Ocular alkali burns recruit neutrophils and triggers neutrophil extracellular trap (NET)-neovascularization cascade effects that limit ocular surface reconstruction and functional repair. However, effective inhibition of the release of neutrophil extracellular traps after a corneal chemical injury, coordination of intrinsic immunity with corneal repair, and exploration of more effective and non-invasive drug-delivery modes are still urgently needed. Using an in vitro coculture system, we found that an alkaline environment stimulates neutrophils to release NETs, which can be regulated by deoxyribonuclease I (DNase I). Inspired by this, we loaded DNase I, which effectively regulates NETs, onto chitosan nanoparticles and combined them with silk fibroin to construct a composite hydrogel that can sustainably regulate NETs. The hydrogel reduced neutrophil extracellular trap production by 50% and neovascularization by approximately 70% through sustained DNase I release after a corneal alkali burn. The complex hydrogel promotes ocular surface reconstruction by modulating the intrinsic immune-cascade neovascularization effect, providing a new research basis for the construction of nanobiomaterials that modulate pathological neovascularization.

## 1. Introduction

The cornea is the outermost transparent tissue of the eye and provides a clear path for light. Owing to its exposure, it is highly susceptible to various types of injuries, which can lead to complications such as corneal clouding and neovascularization, disrupting visual function and affecting quality of life [1]. Chemical eye burns account for 22.1% of all eye injuries, with alkali burns being more common [2]. Corneal alkali burns are characterized by uncontrolled inflammation and corneal neovascularization following injury [3], which can affect corneal transparency and reduce the patient’s quality of life. The effective control of neovascularization after a chemical injury presents a challenge that has been researched by most ophthalmologists.

Polymorphonuclear neutrophils (PMNs) are closely related with autoimmune diseases, and the precise mechanisms have not been clarified [4]. Recently, neutrophil extracellular traps (NETs), an extracellular structure consisting of web-like, decondensed chromatin fibers bound to granule proteins and released by activated PMNs [5], were found to play an important role in autoimmune responses, tumors, and cardiovascular diseases [6,7,8,9]. In infectious diseases, moderate numbers of NETs can inhibit further pathogen transmission. In the case of aseptic corneal inflammation without pathogen involvement, it is necessary to control the rational production of NETs so that the pathologic damage mediated by them does not interfere with corneal repair. Recently, it has been found that after corneal alkali burns, NETs accumulate in large quantities at the site of the injury, exacerbating the inflammatory response and inducing corneal neovascularization [10,11]. We are still at an early stage in understanding the effects of NETs on corneal neovascularization and its underlying mechanisms. This study confirms the important role played by NETs in corneal alkali burns, and the emergence of NETs provides a new target for diagnosing and treating corneal alkali burns and reducing corneal neovascularization. Deoxyribonuclease I (DNase I) is a nucleic acid endonuclease that is highly sensitive to DNA strands [12]. Because of its ability to disrupt the reticular structure of NETs, it has been used to inhibit NETs in vitro and in various animal models with promising results [13,14,15]. However, multiple administrations are required to maintain its effective action concentration, which increases the risk of infection; additionally, the smooth corneal surface is not easy to adhere to. When coupled with the tear-washing effect, this makes it inconvenient to administer the commonly used liquid dosage form of DNase I. Hydrogels are frequently used as drug carriers [16], and the properties of hydrogel alter with changes in materials. Therefore, the construction of a material that can act on the ocular surface in a sustained manner and modulate NETs presents a challenge that must urgently be solved.

Silk pigments are almost transparent to visible light [17] and have high biocompatibility, mechanical strength, and non-inflammatory reactivity [18], rendering them ideal biomaterials for ophthalmology. However, their use as drug carriers is limited by their large pore size, which makes it easy to release drugs abruptly after loading and difficult to release the drugs in a stable and sustained manner. However, by combining filipins with nanoparticles, their biological properties are greatly enhanced [19]. Chitosan (CS) is a biodegradable polysaccharide with strong hydrophilicity and adhesion properties. It can also stabilize the tear film and increase the contact time between the drug and the cornea, and it is often used as a carrier for ophthalmic drugs [20]. We prepared chitosan nanoparticles (CSNPs) loaded with DNase I, synthesized methacryloylated filipin proteins (SilMAs) to facilitate rapid gel-forming ability in the presence of light, and formed a composite hydrogel with the CSNPs to construct a functional biohydrogel that can reside on the ocular surface and continuously release DNase I (DNase I@CS-SilMA, SCD).

In this study, the physicochemical properties of this composite hydrogel were optimized by setting different ratios of each component of the material. The ability of the SCD hydrogel to continuously inhibit NET formation was verified using an in vitro co-culture system. Finally, a mouse corneal alkali burn model was established to investigate the effectiveness and feasibility of this novel light-curing hydrogel in interfering with the intrinsic immune-cascade pathological neovascularization. The sustained modulation of the intrinsic immune-cascade neovascularization by the SCD hydrogel provides a new idea and research basis for promoting the reconstruction of the ocular surface after corneal chemical injuries.

## 2. Results and Discussion

### 2.1. Preparation and Characterization of DNase I@CS-SilMA

**Figure 1:**
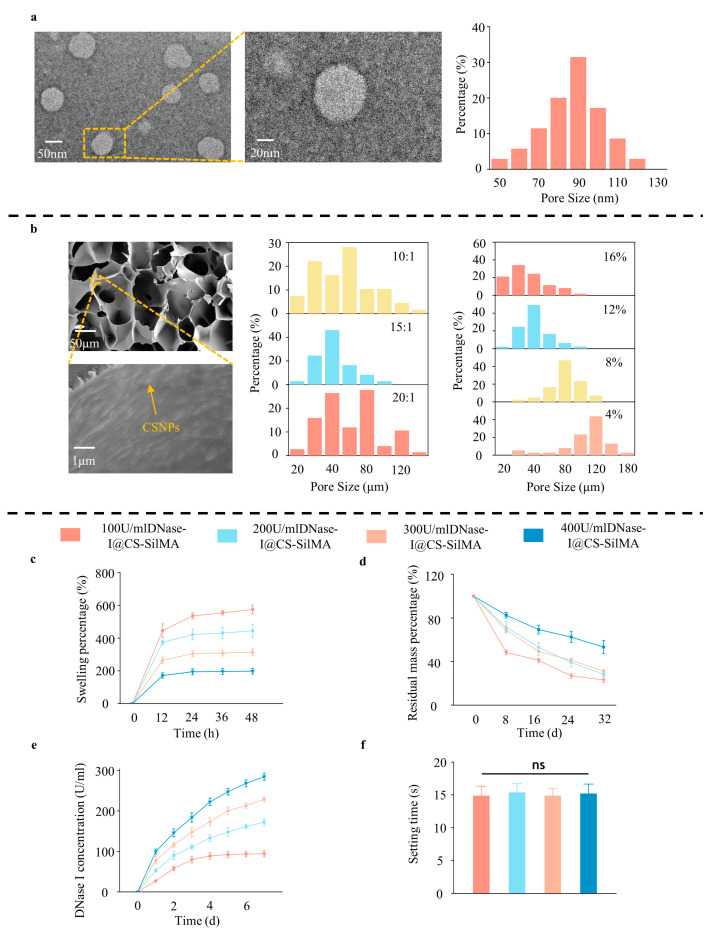
Material construction and properties: (**a**) transmission electron microscopy (TEM) images and size distribution of chitosan-DNase I nanoparticles; (**b**) scanning electron microscopy (SEM) images of SCD indicating the successful binding of CSNPs to SilMA, different ratios of synthesized methacryloylated filipin proteins (SilMA), and chitosan (CS) pore size distribution; (**c**–**f**) dissolution, degradation, release curves, and gel formation times of different DNase I contents. (*n* = 3, all data are expressed as mean ± standard deviation; ns, not significant; groups were compared using one-way ANOVA and Tukey’s test).

Transmission electron microscopy (TEM) photography of DNase I@CSNPs demonstrated the formation of CSNPs loaded with DNase I, with a uniform pore size and an average diameter of 81.18 ± 14.35 nm (Figure 1a). The average pore size of biomaterials is closely related to cell viability and biocompatibility, and pore sizes around 100 μm are considered to be the most suitable for human surface-tissue engineering material science [21,22]. Silk pigment has high biocompatibility and is transparent to visible light, which makes it an effective medical material; however, its pore size is coarse and varied, and it can easily fracture and degrade. After adding nanoparticles, the spatial conformation of the lamellar structure is suppressed, the pores become more orderly, and the physicochemical properties are strengthened [23]. Accordingly, we combined CSNPs loaded with DNase I with SilMA and set different ratios and concentration gradients for the two, and the pore size was found to be relatively concentrated at 80 μm at 0.53% CSNPs/8% SilMA and 120 μm at 0.27% CSNPs/4% SilMA, which is suitable for the construction of ocular surface materials. However, when the SilMA concentration was 4%, the hydrogel-forming time was more than 10 min, which is inconvenient once the hydrogel is used clinically. Therefore, 0.53% CSNPs/8% SilMA was selected in the following test (Figure 1b). Meanwhile, in scanning electron microscopy (SEM) images, the surface of SilMA, which was supposed to be smooth, was filled with small uneven particles, thus demonstrating the successful binding of CSNPs to SilMA (Figure 1b).

To further investigate the pharmacokinetic properties of SCD hydrogels, release, swelling, degradation, and gel-forming assays were performed (Figure 1c–f). The SCD hydrogel swelling and release profiles varied with the DNase I content. The sequence of water absorption was 400 < 300 < 200 < 100 U/mL, while that of the degradation was 100 > 300 > 200 > 400 U/mL. In previous studies, 100 U/mL DNase I was frequently used as an effective measure for inhibiting NETs [11], and the soaking liquid of the SCD with 400 U/mL DNase I could reach 100 U/mL within 24 h, which rapidly inhibited NET generation. In the gel formation experiment, the DNase I content exerted no significant effect on the gel formation time. Considering the ability for body fluid exchange between the hydrogel and the external environment, the integrity-holding properties of the composite system, and the ability to rapidly suppress NETs, 400 U/mL DNase I@0.53%CS-8%SilMA was selected for further research.

### 2.2. SCD Biocompatibility Test

**Figure 2:**
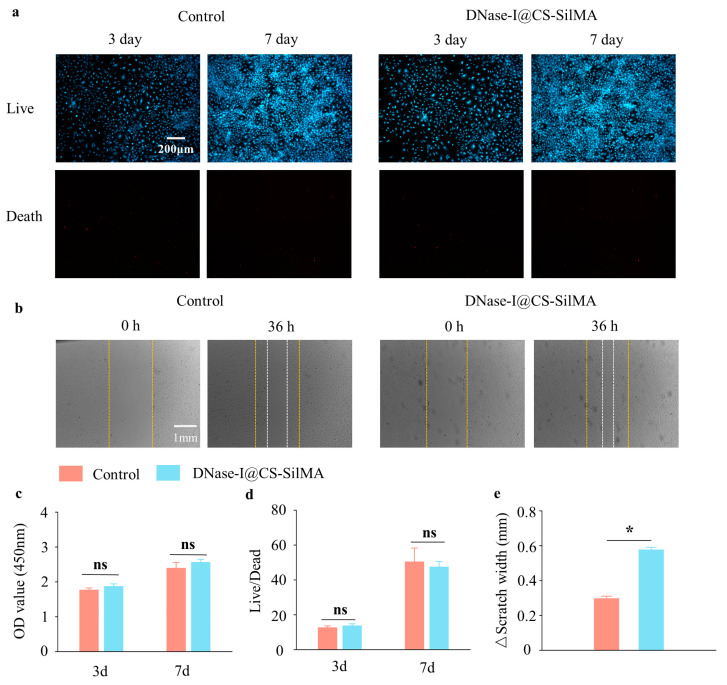
Material biocompatibility: (**a**) live-dead staining of gels and cells cocultured after 3 and 7 days; (**b**) scratch experiment after 36 h: the yellow line was the initial scratch location and the white line was the final cell location; (**c**) semiquantitative analysis of CCK-8 assay and live-dead staining after 3 and 7 days, quantitative analysis of cell migration distance in the scratch assay; (**d**) semi-quantification of live-dead staining; (**e**) semi-quantification of scratch experiment. (*n* = 3, all data are expressed as mean ± standard deviation; * *p* < 0.05; ns, not significant; groups were compared using one-way ANOVA and Tukey’s test).

As shown in Figure 2, corneal stromal cells exhibited normal morphology on the surface of the hydrogel composite system and proliferated normally. Double staining of live/dead cells (Figure 2a,d) and the results of the CCK-8 assay (Figure 2c) revealed that the hydrogel composite system did not significantly affect the proliferation of corneal stromal cells compared to the control group and did not accelerate their death, indicating strong biocompatibility. This may be related to the high biocompatibility of chitosan and silk [21,23]. In the scratch test, 36 h after scratching, the cells moved 0.58 ± 0.01 mm in the SCD group and 0.297 ± 0.01 mm in the control group (Figure 2b,e). As described in previous studies, the primary form of progress yielded by using artificial materials to promote wound healing is to accelerate the process of cell migration and re-epithelialization [24]. The strong abilities of SCD in biocompatibility and cell migration indicate that this material has the potential to accelerate wound repair.

### 2.3. In Vitro Tests of SCD Inhibition of NETs

**Figure 3:**
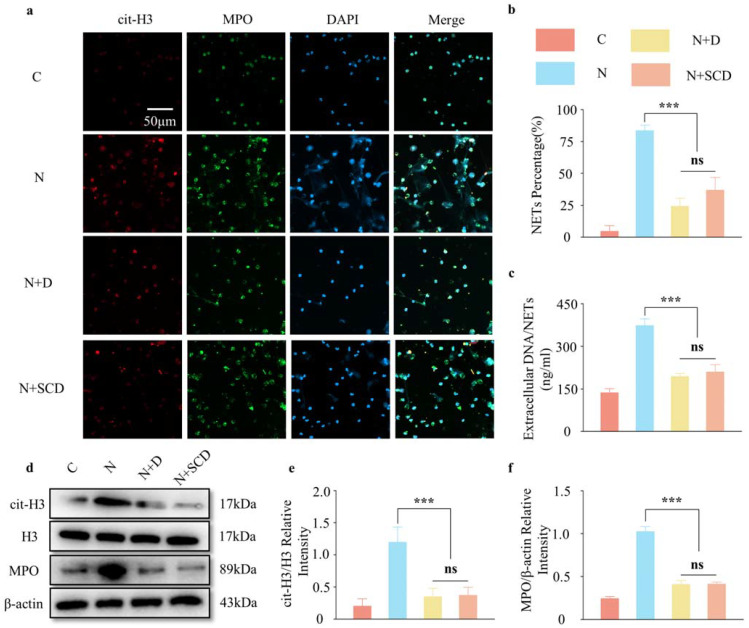
Materials to inhibit neutrophil extracellular traps (NETs) in vitro: (**a**) immunofluorescence of DNase I@CS-SilMA (SCD) co-cultured with NETs; (**b**) immunofluorescence of NET generation-rate statistics; (**c**) NET marker cf-DNA content in the culture medium; (**d**–**f**) Western blot (WB) analysis and semi-quantification of NET markers cit-H3 and MPO. (*n* = 3, all data are expressed as mean ± standard deviation; *** *p* < 0.001; ns, not significant; groups were compared using one-way ANOVA and Tukey’s test).

NaOH was applied in vitro to simulate the microenvironment of corneal alkali burns and stimulate the generation of NETs from PMN, and the ability to inhibit NETs was explored using a 100 U/mL DNase I solution and a one-week leachate of the hydrogel complex. Immunofluorescence results showed that the addition of NaOH (Group N) stimulated the release of large numbers of NETs from PMNs compared with the control (C) group. The addition of either DNase I or SCD composite hydrogel leachate inhibited this trend (with an inhibition efficiency of approximately 60–70%) (Figure 3a,b), and there was no statistically significant difference between the two NET inhibitors (the NET percentage was 24.43 ± 5.10% in the DNase I group and 36.81 ± 8.14% in the SCD group, *p* > 0.05). To make the results more credible, we collected cell culture medium supernatants for the quantitative detection of the NET marker cf-DNA and cell-extracted proteins for the Western blot detection of the NET markers MPO and cit-H3. These results suggested that NaOH could stimulate PMN to release a large number of NETs, and the SCD composite hydrogel could continuously inhibit the release of NETs from NaOH; the effect was comparable to that of fresh DNase I (Figure 3). Although there were differences between the results of multiple parallel experiments, they all indicated that the release of NETs from PMN could be inhibited by the SCD composite hydrogel (Figure 3c–f). However, the ocular surface after corneal alkali burns is a complex and dynamically changing microenvironment, and the inhibition of NET production by SCP and the consequent reduction in corneal neovascularization need to be further explored in vivo.

### 2.4. In Vivo Test of SCD Inhibition of NETs

The dysregulation of the physiological homeostasis of the ocular surface and the inflammatory microenvironment of the cornea that are induced by alkali burns result in persistent visual impairment [25]. As important cells mediating the immune response, neutrophils arrive at the site of injury as early as 6 h after injury [26]. As a novel effector mechanism of neutrophils identified in recent studies, NETs are involved in the onset and progression of a variety of diseases, including autoimmune diseases, dysfunctional wound healing, and sterile inflammation [27,28]. Recent studies have shown that neutrophils release large numbers of NETs after corneal alkali burns [11].

**Figure 4:**
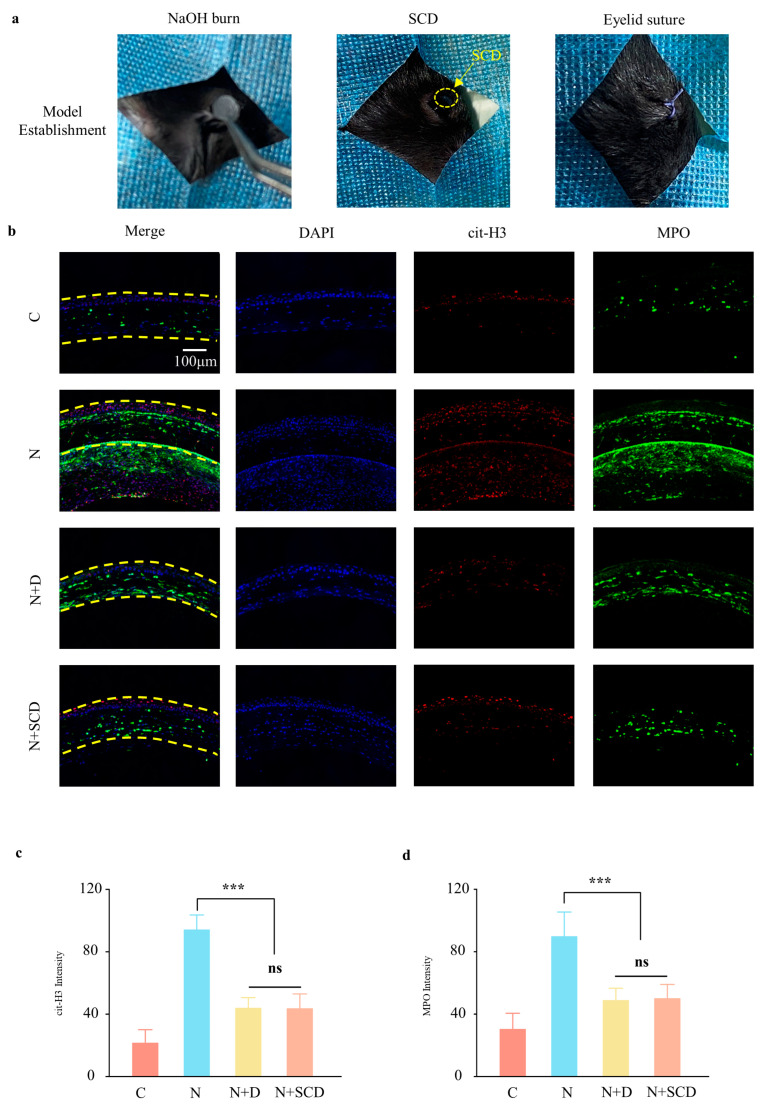
SCD inhibits NETs in a corneal alkali burn model: (**a**) corneal alkali burn model construction and SCD implantation; (**b**) immunofluorescence of corneal cit-H3 and MPO in different groups 7 days after corneal alkali burn; (**c**,**d**) semi-quantitative immunofluorescence analysis of C (Control), N (NaOH), N + D (NaOH + Dnase I), and N + SCD (NaOH + Dnase I@CS-SilMA) groups. (*n* = 3, all data are expressed as mean ± standard deviation; *** *p* < 0.001; ns, not significant; groups were compared using one-way ANOVA and Tukey’s test).

In our in vitro study, we demonstrated that NaOH induces NET formation and established that the SCD slow-release DNase I system regulates NET formation. To investigate the ability of SCD to inhibit NET generation after corneal alkali burns in an in vivo local immune microenvironment, we established a mouse model of corneal alkali burns (Figure 4a). On the seventh day after injury, immunofluorescence staining of the mice’s corneal sections was performed, and the cit-H3 and MPO were more obvious in Group N than Group C, indicating the formation of NETs after corneal alkali burns. Additionally, the expression of NET markers decreased in Group N + D and N + SCD, suggesting the successful inhibition of NETs by DNase I eye drops and SCD (Figure 4b). A semi-quantitative analysis of immunofluorescence data was also performed, and the results suggested a 2.96-fold increase in MPO fluorescence intensity after NaOH stimulation (179.47 ± 25.61 vs. 60.68 ± 16.66, *p* < 0.05), while the fluorescence intensity of another NET marker, cit-H3, increased 4.37-fold (95.07 ± 7.76 vs. 21.52 ± 6.91, *p* < 0.05). The MPO and cit-H3 fluorescence intensities decreased after six daily drops of DNase I or a single application of the SCD hydrogel; however, the difference between them was not statistically significant.

The results of the in vivo test suggest that in the complex immune microenvironment after corneal alkali burns, the SCD group can sustain the release of DNase I and inhibit the production of NETs with only a single application, compared to the eye drop evaluation rate of six times per day in the DNase I solution group. However, carrying bottles of eye drops and using them frequently in a safe and clean environment in daily life is inconvenient for patients. A single application of SCD may improve patient compliance and corneal alkali burn healing outcomes.

### 2.5. In Vivo Test of Neovascularization Inhibition by SCD

The normal cornea is free of vascular invasion due to the relative balance between angiogenic and antiangiogenic factors. When the cornea is subjected to trauma, inflammation, hypoxia, and corneal limbal stem cell deficiency [29], the balance between the two types of factors is disrupted, prompting the formation of corneal neovascularization [30]. Corneal neovascularization is one of the distinguishing features of corneal alkali burns and is one of the main factors leading to vision loss in patients with alkali burns because of the immature vasculature and lack of structural integrity, which predispose the cornea to lipid exudation, inflammation, and scarring. Currently, there are myriad treatment modalities for corneal neovascularization, and a safe and reliable treatment modality is still lacking. Recent studies have found that NET accumulation aggravates neovascularization after corneal alkali burns [10], providing a new direction to address neovascularization after corneal injuries.

**Figure 5:**
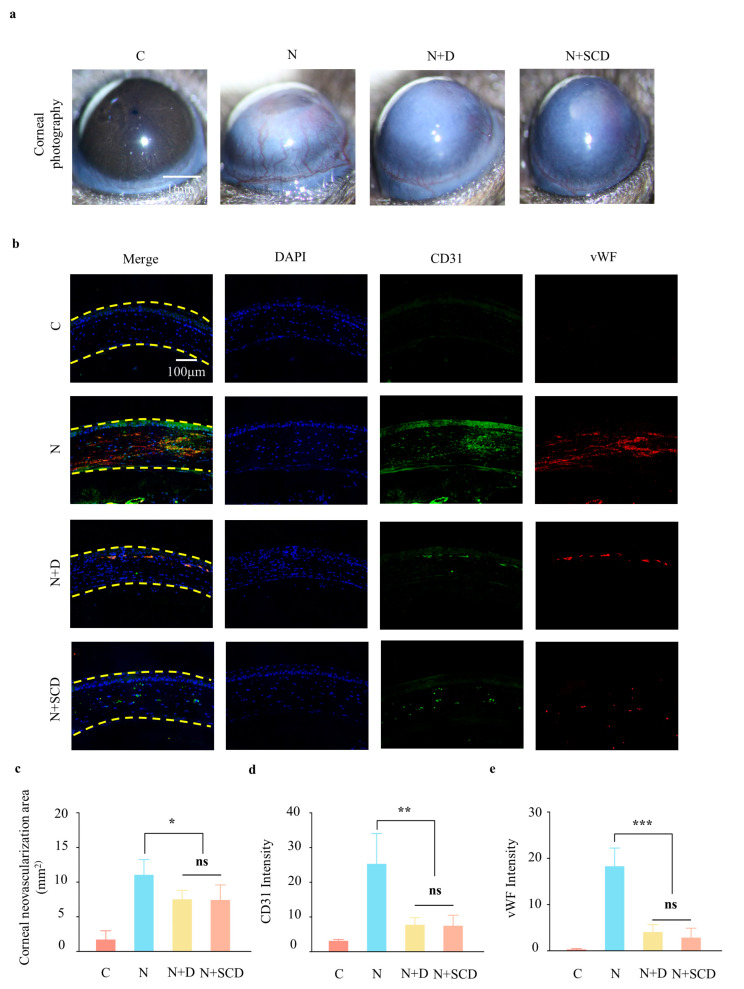
SCD inhibits neovascularization in an animal model: (**a**,**c**) corneal photography assessing the area of neovascularization on the corneal surface; (**b**) immunofluorescence of corneal CD31 and vWF in different groups 7 days after corneal alkali burn; (**d**,**e**) semi-quantitative immunofluorescence analysis of the C (Control), N (NaOH), N + D (NaOH + Dnase I), and N + SCD (NaOH + Dnase I@CS-SilMA) groups. (*n* = 3, all data are expressed as mean ± standard deviation; * *p* < 0.05; ** *p* < 0.05; *** *p* < 0.001; ns, not significant; groups were compared using one-way ANOVA and Tukey’s test).

To investigate the in vivo inhibitory effect of SCD on corneal neovascularization in the NET cascade, we collected photographs of the anterior segment of the eye on day 7 post-injury in an alkali burn model using mice for a quantitative analysis of the neovascularization area (Figure 5a); this revealed that, similar to the addition of droplets of DNase I, the corneal neovascularization surface was approximately only 67% of that stimulated by NaOH alone after the application of SCD, with the difference not being statistically significant (Figure 5c). CD 31 and vWF are commonly used vascular endothelial markers, and immunofluorescence assays of corneal neovascularization were conducted (Figure 5b) [31]. After NaOH stimulation, the expression of corneal neovascularization markers increased and reduced with DNase I or SCD. Moreover, semi-quantitative analysis was performed, and the result was consistent with the immunofluorescence sections (Figure 5d,e), indicating the important role of NETs in inducing corneal neovascularization after corneal alkali burns and the effectiveness of the novel filipin-chitosan nanoscale-loaded deoxyribonuclease particle hydrogel in modulating the NET cascade corneal neovascularization.

## 3. Conclusions

In the present study, we verified that PMNs can release NETs in an in vitro alkaline environment, and based on the important role of NETs in stimulating neovascularization after corneal alkali burns, we constructed a novel filipin chitosan nanoparticle hydrogel loaded with deoxyribonucleic acid enzymes (SCD) and conducted a preliminary exploration of its physicochemical properties, biocompatibility, and ability to regulate NETs as a biomaterial. By optimizing different components of the hydrogel, we constructed SCD that can reach the effective concentration of DNase I within 24 h and continue to act for at least 7 days. During in vitro co-culture experiments, this material exhibited strong biocompatibility and promoted the migration of corneal stromal cells, which accelerated wound repair. Because the injury site of corneal alkali burns is a complex and variable microimmune environment, a mouse alkali burn model was established to better validate the ability of SCD to modulate NET cascade neovascularization in the immune microenvironment after corneal alkali burns. Although the precise mechanism of the NET neovascularization cascade effects remains to be further explored, SCD successfully inhibits corneal neovascularization after alkali burns. In this study, a novel filipin chitosan nanoparticle hydrogel loaded with deoxyribonuclease, constructed based on the NET cascade neovascularization effect after corneal alkali burns, has provided a new theoretical basis and solution to improve visual function and reduce ocular complications after corneal chemical injuries.

## 4. Materials and Methods

### 4.1. Ethics Statement

This study was conducted in accordance with the Declaration of Helsinki, and the protocol was approved by the Ethics Committee of Changzhou Third People’s Hospital (project identification code: 2022-102).

### 4.2. Cell Culture

Neutrophils were extracted using the Animal Bone Marrow Neutrophil Extraction Kit (Solarbio, Beijing, China) and used immediately after resuspension in RPMI-1640 (Corning, New York, NY, USA) supplemented with 2% fetal bovine serum (FBS, Gibco, New York, NY, USA).

NETs were isolated and described as follows: the isolated human neutrophils were seeded into plates pre-coated with poly-L-lysine (PLL). NET formation was stimulated with 5 mM NaOH for 0.5 h. To control the time of NaOH stimulation of neutrophils more accurately and to reduce the influence of NaOH on the NET supernatant, 5 mM HCL was used to neutralize NaOH after stimulation with 5 mM NaOH. A DNase I concentration of 100 U/mL (Sigma-Aldrich, St. Louis, MI, USA) SCD was taken for one week of leachate, and all groups were stored at 37 °C in a temperature chamber for 0.5 h for subsequent experiments.

SCD hydrogels were prepared as described in 4.5 h and were soaked in RPMI-1640 medium at 37 °C in a shaker. The medium was collected as leachate after one week.

Neutrophils in the control group (C) were cultured in RPMI-1640, and the NaOH group (N) was added. Similarly, the medium in the NaOH + DNase I group (N + D) was supplemented with NaOH and DNase I, and that in the NaOH + SCD group (N + SCD) was composed of leachate and NaOH. The concentrations of each component were determined as described above.

After the intervention, the culture supernatant was collected, centrifuged at 3000 rpm for 10 min, and assayed for cf-DNA. The cf-DNA was quantified by applying a green fluorescent dye conjugated to DNA, and the fluorescence intensity was read at an excitation wavelength of 485 nm using a multifunctional enzyme-labeling instrument (Varioskan LUX, Thermo Fisher, Waltham, MA, USA). Standard curves were analyzed and plotted by measuring the fluorescence intensity of calf thymus DNA (Sigma-Aldrich, St. Louis, MI, USA) at the indicated concentrations. Based on the above co-culture system, cellular proteins were extracted by adding RIPA lysate containing a protease inhibitor, and the protein concentration was quantified using a BCA protein quantification kit (Solarbio, Beijing, China). The protein expression of H3cit (ab219407), H3 (ab1971), MPO (ab208670),-actin(ab8227) (Abcam, Cambridge, England) was measured using Western blotting. After electrophoresis, membrane transfer, blocking, and incubation with primary and secondary antibodies, the gels were scanned and photographed using a gel imaging system. The grayscale values of the bands in the images were quantified using ImageJ software.

### 4.3. Immunofluorescence Detection of cit-H3 and MPO

PMNs were extracted and treated as described in Section 4.2, fixed in 4% paraformaldehyde, and permeabilized with 0.3% Triton-X 100 (Sigma-Aldrich, St. Louis, MI, USA). They were then incubated overnight with 5% bovine serum albumin (BSA; Biosharp, Hangzhou, China) and the primary antibodies against cit-H3 (Abcam, Waltham, MA, USA) and MPO (Abcam, Waltham, MA, USA), washed, and then incubated with secondary antibodies at room temperature for 2 h. DAPI (Abcam, Waltham, MA, USA) was used for staining, and the cells were observed under an inverted fluorescence microscope. Semi-quantitative fluorescence analysis was performed using ImageJ software.

### 4.4. Construction of Corneal Alkali Burns Animal Models

Wild-type C57BL/6J (WT) mice were purchased from JOINN Laboratories (Suzhou, China). All mice were 7–9-week-old males weighing 22–26 g. A mouse corneal alkali burn model was constructed as described previously [32]. Briefly, 1% pentobarbital sodium (40–50 mg/kg) was injected intraperitoneally, and corneal drops of 0.5% proparacaine were added. Filter paper (2 mm diameter) soaked in 0.1 mol/L NaOH solution was placed in the cornea of the right eyes of the mice for 40 s. Afterward, the eyes were thoroughly rinsed with sterile PBS for 2 min, and different ocular interventions were applied to the groups. In the DNase I group, 100 U/mL DNase I solution was dropped into the eyes of the mice six times a day, and the mice in the SCD group received SCD hydrogel overlaid on the cornea. To avoid eye scratching, the eyelids were sutured using hydrogel. The anterior segments of the eyes were photographed and corneal sampling was performed on day 7.

### 4.5. Preparation of Composite Hydrogels with DNase I-Loaded Silk Chitosan Nanoparticles (SCD Ratios for 400 U/mL DNase-I@0.53%CS-8%SilMA as an Example)

Chitosan (53 mg, 200–400 mPa·s) was dissolved in 10 mL of 1% (*w*/*v*) acetic acid solution under magnetic stirring at room temperature. When the solution was clear and transparent, the pH was adjusted to 6.0 with 10 M NaOH solution and filtered three times with a 0.45 μm filter membrane (to prepare the DNase I-CS, 4000 U DNase I (Aladdin, Shanghai, China) was resuspended in CS nanoparticles). An aqueous solution of tripolyphosphate (TPP) (0.7 mg/mL) was added dropwise under magnetic stirring at 720 rpm to achieve a CS/TPP mass ratio of 4:1. At this point, the solution produced a transparent opalescent color, indicating that CS and TPP had undergone ion crosslinking polymerization. At this point, the chitosan nanoparticle solution loaded with DNase I was prepared. Lithium phenyl (2,4,6-trimethylbenzoyl) phosphinate (LAP) and 800 mg SilMA were added and dissolved at room temperature for 1 h, during which the solution was stirred and shaken several times. The resulting solution was stored.

### 4.6. SCD Composite Hydrogel Characterization

TEM was used to photograph the chitosan nanoparticles, and SEM was used to photograph the freeze-dried SCD hydrogels for nanoparticle diameter and hydrogel pore-size analysis. The in vitro release of DNase I was evaluated using a DNase I Assay Kit (Beyotime, Shanghai, China). The in vitro degradation and water absorption of the composite system were analyzed by weighing. The gel formation times of the SCD replica hydrogels with different DNase I contents were determined under UV irradiation; each group was measured six times, and the average value was recorded.

### 4.7. SCD Composite Hydrogel Biocompatibility Testing

Corneal stromal cells with or without SCD hydrogel were placed in DMEM medium and co-cultured in a 37 °C incubator, and after the cells were stabilized, 200 µL of gun tip was applied to straighten the line, and photographs were taken at 0 h and 36 h, and three fields of view were taken from each group of cells to take measurements of the migration distance of the cells. A live/dead staining assay (Invitrogen, Waltham, CA, USA) was then performed. After 3 and 7 days of co-culture, live/dead kits were added to the plates and incubated for 30 min at room temperature. The results were obtained using an inverted fluorescence microscope. CCK-8 (Dojindo, Kumamoto-ken, Japan) was also added to the medium on days 3 and 7, and 100 μL was placed in a 96-well plate after 4 h; the absorbance was measured at 450 nm using an enzyme marker.

### 4.8. Anterior Segment Photography

After intraperitoneal injection of 1% pentobarbital sodium (40–50 mg/kg), color photographs of mouse corneas were recorded using a slit-lamp-mounted digital system (SL-D Digital Slit Lamp, Topcon, Tokyo, Japan). The entire cornea was imaged using a 10-fold magnification of the region of interest. Illumination was performed using a 45° angled beam on a slit-lamp biomicroscope with a diffuser filter and a variable flash intensity.

The anterior images were independently analyzed by two observers. The corneal neovascularization area was selected and analyzed by the observers using ImageJ software. The average of the observers was considered the final area.

### 4.9. Immunofluorescence Staining

Whole corneal sections were used to assess corneal NETs and neovascularization. The eyeballs were then removed and embedded in paraffin. Slices were made perpendicular to the cornea along the center of the cornea, and the thickness of the slices was about 5 μm. Immunohistochemical staining for cit-H3 (Abcam, Waltham, MA, USA), MPO (Abcam, Waltham, MA, USA), vWF (Abcam, Waltham, MA, USA), and CD31 (CST, Boston, MA, USA) fluorescence was also performed on the sections for comparison with the control group.

### 4.10. Statistical Analysis

All experiments were performed at least in triplicate, and the data are presented as the standard deviation. One-way analysis of variance (ANOVA) was used to compare means using Tukey’s post hoc test. *p*-values of 0.05, 0.01, and 0.001 were used as the thresholds.

## Data Availability

Data can be obtained from Peirong Lu (lupeirong@suda.edu.cn).
